# 2021 global burden of disease study: a comprehensive analysis of glaucoma in the middle-aged and older adult population at global, regional, and national levels

**DOI:** 10.3389/fpubh.2025.1526061

**Published:** 2025-11-11

**Authors:** Xiang Li, Guo-xin Huang, Ran Li, Zhi-Jie Zhang, Si-Qi Zhang, Lei Zhu, Jia-feng Tang, Jia-qi Wang, Xiong Wang

**Affiliations:** 1 Department of Anesthesiology, Luzhou Maternal and Child Health Hospital (Luzhou Second People's Hospital), Luzhou, Sichuan, China; 2 Chongqing Key Laboratory of Development and Utilization of Genuine Medicinal Materials in Three Gorges Reservoir Area, Chongqing Three Gorges Medical College, Chongqing, Wanzhou, China; 3 Department of Evidence-Based Medicine Center, Xiang yang No.1 People’s Hospital, Hubei University of Medicine, Xiangyang, China; 4 Department of Immunology, Key Laboratory of Human Functional Genomics of Jiangsu Province, Nanjing Medical University, Nanjing, China; 5 Department of Clinical Laboratory, Xiang yang No.1 People’s Hospital, Hubei University of Medicine, Xiangyang, China; 6 Department of Ophthalmology, Xiang yang No.1 People’s Hospital, Hubei University of Medicine, Xiangyang, China

**Keywords:** GBD 2021, eye disease, glaucoma, disease burden, global disease burden

## Abstract

**Background:**

Glaucoma is a leading cause of irreversible blindness globally, with a particularly significant impact on middle-aged and older adults (aged 45 years and above), substantially affecting their quality of life and imposing considerable socio-economic burdens. Comprehensive assessments of the burden of glaucoma in this age group at global, regional, and national levels are crucial for shaping health policies and optimizing resource allocation.

**Methods:**

Data on the burden of glaucoma among individuals aged 45 years and above were obtained from the 2021 Global Burden of Disease (GBD) study. The Average Annual Percent Change (AAPC) was used to evaluate trends in glaucoma burden from 1990 to 2021 among this population. The Slope Index of Inequality (SII) and the Concentration Index (CI) were employed to analyze both absolute and relative health inequalities in the burden of glaucoma. An Age–Period–Cohort model focusing on the 45+ age group was fitted using the NORDPRED package to predict the future burden of glaucoma. Additionally, frontier analysis was conducted to assess the relationship between the burden of glaucoma and socio-demographic development in the older adult population, using non-parametric Data Envelopment Analysis (DEA) to define a boundary based on the level of development for the minimal achievable burden.

**Results:**

In 2021, there were 5.34 million cases of glaucoma among the global population aged 45 years and above (95% UI: 4.22–6.45 million), with an age-standardized prevalence rate (ASPR) of 889.3 per 100,000 population (95% UI: 700.2–1078.5). The AAPC for glaucoma in this age group from 1990 to 2021 was −0.82 (95% CI: −0.85 to −0.8), indicating a downward trend in both ASPR and age-standardized DALY rate (ASDR) globally among middle-aged and older adults. At the regional level, areas with lower Socio-Demographic Index (SDI) exhibited higher ASPRs, whereas high SDI regions recorded lower rates. Nationally, countries such as Niger and Nigeria demonstrated the highest age-standardized rates within this demographic. Health inequality analyses revealed that countries with lower SDI bear a disproportionately higher burden of glaucoma among middle-aged and older adults. Predictions indicate that although the number of global cases in this age group may rise, the overall burden of glaucoma is expected to gradually decline.

**Conclusion:**

As one of the leading causes of blindness among middle-aged and older adults worldwide, glaucoma remains a significant public health concern. Although the absolute number of cases in individuals aged 45 years and above continues to rise due to population growth and aging, both the ASPR and ASDR have shown declining trends. This decline reflects meaningful progress in the prevention, diagnosis, and management of the disease. To address the ongoing increase in case numbers, targeted policy interventions are needed to ensure effective prevention and management strategies, contributing to the achievement of global sustainable development goals. Furthermore, frontier analysis identifying disparities between national development levels and the burden of glaucoma in the older adult population can aid in optimizing the allocation of global health resources.

## 1 Introduction

Glaucoma is a neurodegenerative eye disease and a leading cause of irreversible blindness worldwide. It is marked by the degeneration of the optic nerve and the loss of retinal ganglion cells, which leads to significant visual field loss (1). The development and severity of glaucoma largely depend on the optic nerve’s ability to withstand pressure-induced damage (2). Beyond directly affecting vision, glaucoma imposes significant lifestyle restrictions on patients, increasing the risks of falls and driving difficulties. Along with treatment costs, it imposes substantial psychological and economic burdens on patients and their families (2). The impact of glaucoma is particularly severe in middle-aged and older adults, as the risk of developing the disease significantly increases with age (3, 4). The global trend towards an aging population highlights this issue, especially in high-income and rapidly developing countries (5).

Recent studies have shown that the prevalence of glaucoma in the older adults can be as high as 3.5%, a rate that increases with age. This affects individuals’ quality of life, particularly in vision-critical daily activities and social interactions, and also exacerbates the social and economic burden (6).

The Global Burden of Disease (GBD) Collaborative Network initiated the GBD study, which assesses 288 causes of death, 371 diseases and injuries, and 88 risk factors across 204 countries and regions (7). This study on disease burden serves as a crucial foundation for the development and implementation of health policies. Consequently, countries worldwide have been analyzing disease burden to make informed, scientifically-based health decisions.

The GBD 2021 study stands as the most detailed and authoritative resource for assessing the global impact of all diseases and injuries, providing insights into health deterioration trends and levels worldwide. This pivotal tool measures life loss due to preventable conditions and, for the first time, quantifies health impacts attributable to the COVID-19 pandemic (7). The burden of glaucoma is strongly associated with socio-economic status, age, gender, and various other factors. According to the 2019 World Health Organization (WHO) Vision Report, 76 million people aged 40–80 globally were projected to have glaucoma in 2020, up from 69.2 million in 2016, with the number expected to reach 95.4 million by 2030. However, with changes in lifestyles and increased life expectancy, the incidence pattern of glaucoma has also changed. Following the COVID-19 pandemic, there remains a significant gap in global burden reports on glaucoma based on the latest GBD study. Focusing on the middle-aged and older population is particularly critical, as these groups face a higher risk of glaucoma, which is further exacerbated by aging populations globally. By honing in on individuals aged 45 and older, this research seeks to uncover specific risk factors and trends that can inform more effective prevention and management strategies tailored to this vulnerable group, ultimately contributing to better health outcomes and reduced disparities in eye health care.

This article describes the latest epidemiology of glaucoma after the COVID-19 pandemic. It analyzes the prevalence and Disability-Adjusted Life Years (DALYs) of glaucoma globally, regionally, and nationally from 1990 to 2021, and provides predictions for the future of glaucoma.

## 2 Methods

### 2.1 Data sources

Glaucoma is defined according to the International Classification of Diseases, 10th Revision (ICD-10 codes: H40-H40.6 and H40.8-H40.9). For our analysis, we selected the population aged 45^+^. The GBD 2021 study provides estimates of DALYs and the prevalence of glaucoma based on both crude and age-standardized values. These data are publicly available from the Institute for Health Metrics and Evaluation (IHME).^1^ The GBD Collaborative Network covers 204 countries and territories across all major world regions, ensuring comprehensive global coverage for disease burden estimates. Previous publications have detailed the specifics and usage of this database (8).

The GBD annually estimates metrics such as prevalence, incidence, mortality, and DALYs. DALYs are determined by adding years of life lost to premature mortality to years lived with disability. This measure extends beyond mere prevalence and mortality rates to more comprehensively represent the total human suffering associated with a disease (9).

The SDI (Socio-Demographic Index) is a predictive indicator that describes a country’s socio-demographic status and shows a strong correlation with its population’s health outcomes (10). The SDI gauges socio-demographic progress by incorporating factors like per capita income, education levels, and total fertility rate (TFR). It scales from 0 to 1, where 0 indicates the lowest income, education levels, and highest TFR, and 1 denotes the highest income, education levels, and lowest TFR. Based on the 2021 SDI assessments, countries and regions are classified into five categories: low, low-middle, middle, high-middle, and high SDI.

## 3 Statistical analysis

In this study, we quantified the prevalence and DALYs of glaucoma using age-standardized rates (ASR) and their 95% UI. The predicted values per 100,000 population, including their 95% UI, were calculated using the world standard population. Age-standardized DALY rates (ASDRs) were calculated using the world standard population as a reference. Specifically, DALY rates for each age group were weighted according to the corresponding proportion in the world standard population, and then summed across all age groups to obtain an internationally comparable rate. We employed the Joinpoint regression analysis model to assess the temporal trends in age-standardized prevalence rates (ASPR) and age-standardized DALYs (ASDR) of glaucoma globally and regionally. This model is commonly used in epidemiological research to identify and characterize significant change points in time series data across global and national scales. We conducted a detailed analysis of the ASPR and ASDR for the years 1990 and 2021. To evaluate the overall trends during this period, we calculated the average annual percentage change (AAPC). An AAPC with a lower limit of the 95% confidence interval (CI) exceeding zero indicates an increasing trend, whereas an AAPC with the upper limit of the 95% UI below zero suggests a decreasing trend. When the 95% UI of the AAPC includes zero, it indicates a stable trend. We elucidate the burden of glaucoma across gender, age groups (spanning every 5 years from 45 to 95+), and SDI. Moreover, we examine the global impact of glaucoma on a national scale and analyze the correlation between socio-demographic status and DALY, using the SDI as a framework.

Moreover, we plotted dual Y-axis graphs for 2021 that report the number and age-standardized prevalence and DALY rates by gender, age, and SDI regions, as well as graphs illustrating the relationship between SDI and changes across countries.

To provide information for policies, planning, and practices aimed at achieving universal health coverage and reducing health inequalities, we used the Slope Index of Inequality (SII) and Concentration Index (CI) to analyze absolute and relative health inequalities in glaucoma (11). The SII was calculated by regressing each country’s DALYs on the SDI-related relative position scale, defined by the midpoint of the cumulative population range of SDI rankings. The CI was determined by calculating the ratio of the area between the Lorenz curve and the diagonal, using cumulative SDI rankings and cumulative DALY fractions. This study also predicted global prevalence and DALYs for 2040 to assess the future GBD of glaucoma. The model was implemented using the NORDPRED package in R. All statistical analyses and visualizations were conducted using R software, with *p* < 0.05 considered statistically significant (12).

We utilized frontier analysis to further assess the relationship between the burden of glaucoma and socio-demographic development. To create a nonlinear boundary representing the minimum achievable burden based on the level of development, we employed non-parametric Data Envelopment Analysis (DEA), as described in prior studies. The distance between a country’s observed DALY rate and this boundary is termed the efficiency gap, indicating the unrealized health gains relative to the country’s or region’s current development level (13, 14).

## 4 Results

### 4.1 The global burden of glaucoma and temporal trends from 1990 to 2021

As indicated in Tables 1, 2, the global number of glaucoma cases increased from 4.07 million (95% UI 3.20–5.13) in 1990 to 7.58 million (95% UI 5.96–9.58) in 2021, an 86.24% rise. Additionally, DALYs increased by 62.51% over the past 32 years, with the ASDR reaching 33.13 per 100,000 population in 2021. Western Sub-Saharan Africa reported the highest ASPR and ASDR among the 21 GBD regions, with rates of 589.5 and 118.9 per 100,000 individuals, respectively. Central Latin America had the lowest ASPR at 141.3 per 100,000, followed by North Africa and the Middle East at 159.7, and South Asia at 183.5. From 1990 to 2021, the AAPC for both ASPR and ASDR was negative across all 21 GBD regions, indicating a decline in these rates over the past 32 years.

**Table 1:** Prevalence and ASPR of glaucoma in 1990 and 2021 and the temporal trends from 1990 to 2021.

Prevalence	1990		2021		1990–2021
Location	Prevalent cases No.(95%UI)	ASPR per 100,000 No.(95%UI)	Prevalent cases No.(95%UI)	ASPR per 100,000 No.(95%UI)	AAPC in ASPR No.(95%Cl)
Global	4072106.5 (3204763.7–5131929.6)	425.5 (335.6–535.0)	7587672.9 (5969840.6–9582253.8)	329.8 (259.7–415.8)	−0.82 (−0.85–0.8)
Andean Latin America	34609.9 (25460.9–46372.2)	709.5 (522.0–950.9)	77363.0 (57941.1–101882.6)	502.5 (376.5–661.6)	−1.11 (−1.16–1.06)
Australasia	13344.7 (10291.1–17134.1)	213.5 (164.7–274.4)	30385.9 (23222.7–39241.0)	183.5 (140.0–237.6)	−0.49 (−0.51–0.46)
Caribbean	42425.9 (31456.2–56624.9)	637.6 (472.6–850.9)	70581.5 (53152.8–92904.1)	475.8 (358.6–626.0)	−0.94 (−0.95–0.92)
Central Asia	55141.1 (40908.6–73518.2)	482.9 (358.7–642.6)	72637.6 (54169.7–96681.2)	387.4 (289.8–512.4)	−0.71 (−0.73–0.69)
Central Europe	67897.4 (51945.2–88736.6)	180.2 (137.7–235.1)	92636.6 (70589.8–120918.5)	141.3 (107.4–185.0)	−0.78 (−0.80–0.76)
Central Latin America	118504.3 (92071.5–151732.9)	616.7 (479.7–788.7)	299310.5 (231536.4–384697.1)	460.9 (357.1–591.4)	−0.94 (−0.95–0.92)
Central Sub-Saharan Africa	25439.1 (19638.7–32932.8)	639.8 (498.2–820.4)	57793.1 (44430.5–74844.2)	589.5 (457.2–757.8)	−0.27 (−0.29–0.25)
East Asia	602900.5 (468910.0–762760.9)	323.2 (253.0–406.9)	1137135.4 (880486.0–1460886.4)	196.7 (152.6–252.0)	−1.67 (−1.92–1.42)
Eastern Europe	226898.0 (179701.0–285404.3)	317.9 (252.0–399.4)	252879.1 (200455.1–318360.7)	253.5 (200.6–318.9)	−0.73 (−0.76–0.70)
Eastern Sub-Saharan Africa	166898.4(132198.0–210420.2)	1072.2(851.7–1348.2)	308159.2(242102.3–390960.3)	866.2(684.0–1093.3)	−0.69(−0.73–0.66)
High-income Asia Pacific	124151.5 (98915.4–154849.8)	245.8 (196.4–305.6)	323089.9 (259696.7–401846.4)	200.7 (160.4–251.6)	−0.66 (−0.69–0.62)
High-income North America	172910.8 (137755.2–215790.8)	170.0 (135.3–212.5)	317228.1 (252016.1–396862.1)	159.7 (126.7–200.1)	−0.20 (−0.27–0.14)
North Africa and Middle East	402591.3 (311505.1–519279.0)	1094.4 (849.6–1405.9)	776896.8 (599920.1–1003360.5)	761.7 (590.4–978.2)	−1.14 (−1.23–1.05)
Oceania	2371.5 (1695.9–3238.5)	411.3 (296.9–558.0)	5139.0 (3735.0–6931.7)	337.6 (246.6–453.3)	−0.64 (−0.70–0.59)
South Asia	803203.7 (627234.1–1017055.1)	642.3 (503.8–810.8)	1621629.5 (1263588.7–2063988.8)	450.8 (352.9–571.3)	−1.12 (−1.28–0.97)
Southeast Asia	211833.9 (161546.7–273941.9)	382.4 (293.2–492.6)	422596.0 (326722.2–541709.5)	272.8 (211.9–348.6)	−1.08 (−1.11–1.05)
Southern Latin America	34689.3 (26149.0–45645.3)	296.8 (223.8–390.2)	58174.5 (43932.4–76498.9)	232.4 (175.4–306.0)	−0.78 (−0.81–0.75)
Southern Sub-Saharan Africa	53767.6 (41906.9–68654.6)	864.8 (676.9–1099.6)	96003.9 (75298.4–121691.1)	740.0 (583.2–933.8)	−0.49 (−0.62–0.35)
Tropical Latin America	163390.8 (130007.9–204138.2)	789.4 (629.1–983.8)	420428.9 (333742.7–527430.4)	621.3 (493.6–778.1)	−0.82 (−1.08–0.55)
Western Europe	493873.2 (379793.1–636670.0)	298.2 (228.8–384.9)	695423.7 (540400.8–888406.0)	223.1 (173.0–286.3)	−0.94 (−0.97–0.91)
Western Sub-Saharan Africa	255263.4 (203230.2–319094.9)	1310.6 (1045.9–1633.6)	452180.6 (356321.3–573021.3)	1077.1 (852.5–1356.9)	−0.63 (−0.67–0.59)

No, Number; ASPR, Age-standardized prevalence rate; AAPC, Average Annual Percentage Change; UI, Uncertainty interval; CI, Confidential interval.

**Table 2:** DALYs and ASDR of glaucoma in 1990 and 2021 and the temporal trends from 1990 to 2021.

DALY	1990		2021		1990–2021
Location	DALYs No.(95%UI)	ASDR per 100,000 No.(95%UI)	DALYs No.(95%UI)	ASDR per 100,000 No.(95%UI)	AAPC in ASDR No.(95%Cl)
Global	467600.4 (306193.8–685253.7)	48.9 (32.1–71.4)	759900.2 (503116.8–1097384.7)	33.1 (21.9–47.8)	−1.24 (−1.26–1.21)
Andean Latin America	4100.8 (2477.0–6353.5)	84.5 (51.2–130.4)	7876.1 (4921.7–11934.4)	51.3 (32.1–77.7)	−1.59 (−1.66–1.53)
Australasia	1430.9 (888.5–2177.9)	22.9 (14.2–34.8)	2963.2 (1829.6–4497.1)	18.0 (11.0–27.5)	−0.77 (−0.81–0.72)
Caribbean	5168.7 (3152.8–8058.8)	77.9 (47.6–121.1)	7636.1 (4756.8–11693.2)	51.4 (32.1–78.7)	−1.33 (−1.35–1.31)
Central Asia	6543.7 (3998.3–10166.9)	57.3 (35.2–88.9)	7852.5 (4833.2–12185.3)	42.1 (26.1–64.8)	−0.99 (−1.01–0.97)
Central Europe	7042.9 (4436.5–10731.1)	18.8 (11.8–28.5)	8485.9 (5444.7–12712.2)	13.0 (8.3–19.5)	−1.19 (−1.23–1.15)
Central Latin America	12452.0 (8034.9–18231.2)	65.2 (42.1–95.2)	26760.2 (17609.0–38622.5)	41.4 (27.2–59.6)	−1.47 (−1.49–1.44)
Central Sub-Saharan Africa	2441.2 (1528.2–3659.6)	62.9 (39.9–92.9)	5088.0 (3240.3–7624.1)	53.1 (34.1–78.7)	−0.55 (−0.58–0.52)
East Asia	69522.8 (44463.0–102551.3)	37.1 (23.9–54.2)	100817.5 (65562.6–148668.6)	17.4 (11.4–25.6)	−2.44 (−2.80–2.08)
Eastern Europe	25309.8 (16410.1–37101.7)	35.6 (23.1–51.9)	24980.6 (16375.9–36065.0)	25.1 (16.4–36.2)	−1.11 (−1.14–1.09)
Eastern Sub-Saharan Africa	21277.4 (13887.1–31148.2)	136.9 (89.8–199.0)	36478.0 (23945.4–53626.6)	103.6 (68.2–151.0)	−0.89 (−0.93–0.86)
High-income Asia Pacific	15017.0 (9827.0–21980.7)	29.3 (19.2–42.8)	34518.4 (22848.2–49614.7)	22.0(14.4–31.9)	−0.92 (−0.97–0.88)
High-income North America	18852.4 (12451.5–27015.2)	18.6 (12.3–26.7)	33183.0 (22128.6–47723.3)	16.7 (11.1–24.1)	−0.34 (−0.41–0.27)
North Africa and Middle East	51076.5 (32648.1–76112.8)	139.1 (89.3–205.5)	85940.4 (55035.0–127192.2)	85.2 (54.9–125.3)	−1.54 (−1.66–1.43)
Oceania	296.5 (171.2–473.5)	51.0 (30.0–80.1)	594.5 (349.9–938.5)	39.0 (23.4–60.8)	−0.85 (−0.98–0.73)
South Asia	85478.3(56076.3–124690.7)	69.4(45.7–100.2)	152206.3 (102191.9–216913.3)	43.0 (29.0–61.1)	−1.53 (−1.77–1.28)
Southeast Asia	22716.8 (14534.5–33640.7)	41.6 (26.7–61.2)	38785.0 (25333.1–56876.4)	25.4 (16.6–37.2)	−1.58 (−1.63–1.53)
Southern Latin America	3675.6 (2221.4–5694.8)	31.5 (19.1–48.8)	5406.6 (3368.2–8149.5)	21.6 (13.4–32.6)	−1.21 (−1.25–1.17)
Southern Sub-Saharan Africa	6904.2 (4423.4–10271.7)	110.5 (71.2–163.3)	11337.9 (7354.4–16660.0)	87.2 (56.9–127.3)	−0.74 (−0.90–0.58)
Tropical Latin America	16693.8 (11082.1–24115.4)	81.7 (54.3–117.5)	38739.4 (26072.9–55274.6)	57.5 (38.7–82.0)	−1.11 (−1.16–1.05)
Western Europe	63124.3 (40191.4–94205.2)	38.2 (24.2–57.1)	81622.9 (52769.8–119830.6)	26.5 (17.1–39.2)	−1.18 (−1.20–1.15)
Western Sub-Saharan Africa	28475.1 (18881.5–41236.9)	149.4 (99.3–215.0)	48627.8 (32076.7–70921.2)	118.9 (78.6–172.2)	−0.73 (−0.82–0.64)


DALYs, Disability-adjusted life-years; No, Number; ASDR, Age-standardized DALY rate; AAPC, Estimated annual percentage change; UI, Uncertainty interval; CI, Confidential interval.

**Figure 1:**
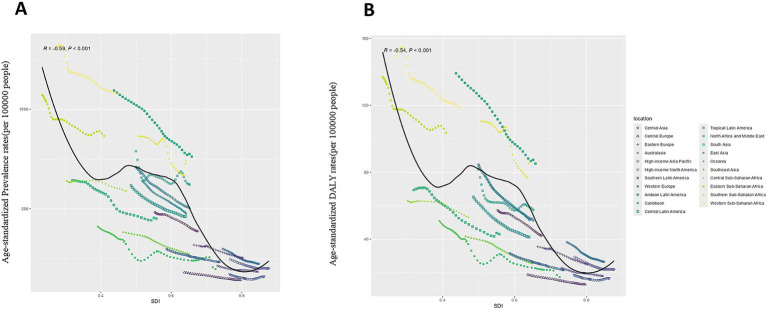
The global disease burden of Glaucoma in GBD regions by the Socio-demographic Index. **(A)** Age-standardized Prevalence Rate: ASPR; **(B)** Age-standardized DALYs Rate: ASDR.

We further examined the relationship between the five SDI tiers and age-standardized rates at the regional level, as shown in Figure 1. Our analysis reveals that regions with lower SDI levels have higher ASPR, while those with higher SDI levels have lower ASPR. Figure 1A demonstrates a significant inverse correlation between SDI and ASPR regionally, a trend that is also evident with ASDR in Figure 1B. These findings indicate that lower-income regions bear a greater burden of glaucoma, highlighting disparities in disease prevalence and DALY rates associated with economic status.

### 4.2 Changes in the burden of glaucoma at the national and regional levels

**Figure 2:**
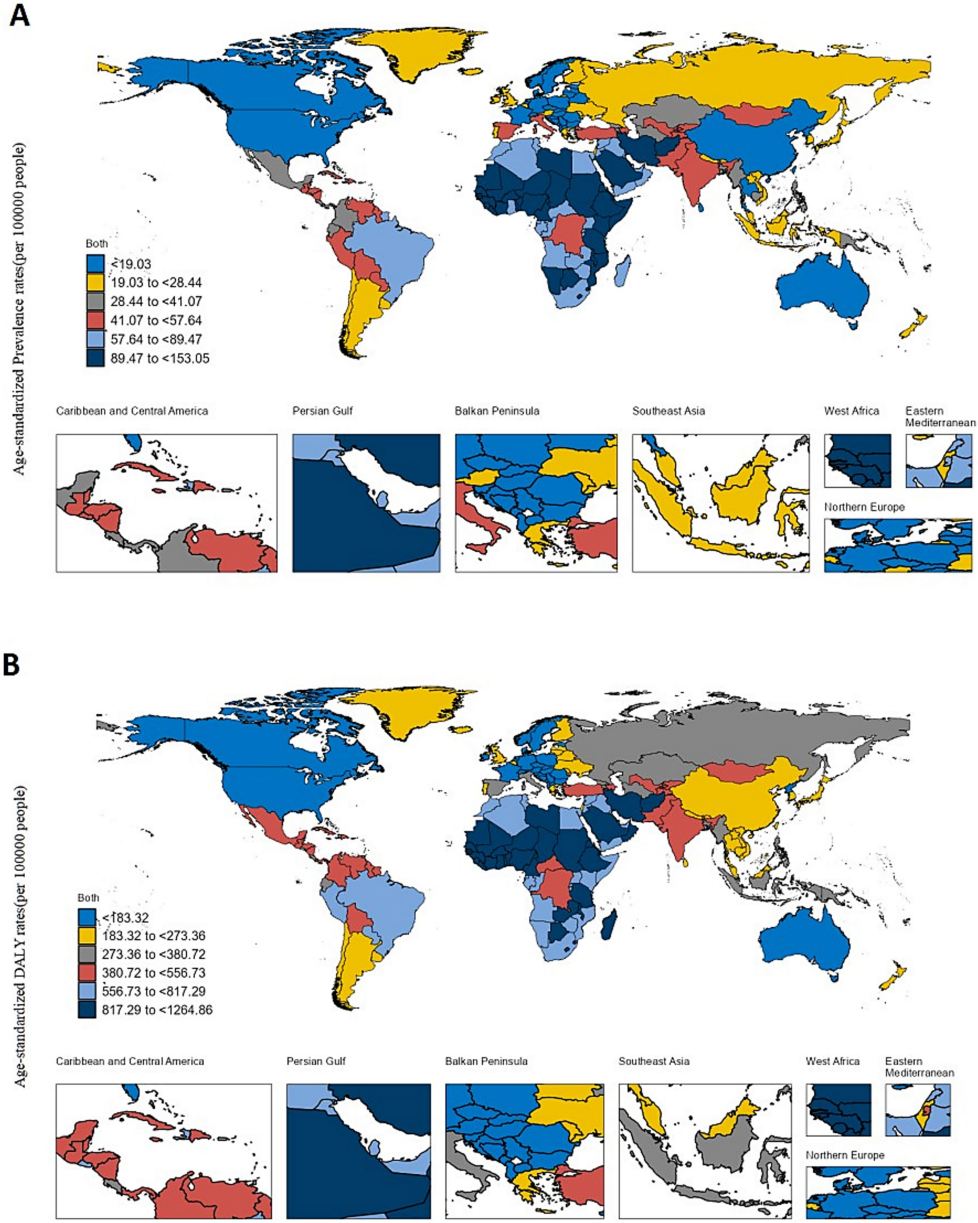
The global disease burden of Glaucoma in 204 countries and territories. **(A)** The ASPR (per 100,000 populations) of Glaucoma in 2021. **(B)** The ASDR (per 100,000 populations) of Glaucoma in 2021.

Figure 2 provides a summary of the national burden of glaucoma. As depicted in Figure 2A, the ASDR varies from 2.15 (95% UI: 1.14–3.70) to 153.05 (95% UI: 93.81–235.31). The highest ASDR are observed in Niger (153.05; 95% UI: 93.81–235.31), followed by Botswana (140.19; 95% UI: 86.34–216.60), Ethiopia (134.84; 95% UI: 87.25–198.01), Iran (133.85; 95% UI: 89.43–191.45), and Nigeria (133.32; 95% UI: 89.07–190.64).

As depicted in Figure 2B, the ASPR varies from 64.51 per 100,000 population (95% UI: 42.97–95.39) to 1264.86 per 100,000 population (95% UI: 950.90–1661.79). The highest ASPR are recorded in Niger (1264.86 per 100,000 population; 95% UI: 950.90–1661.79), followed by Nigeria (1174.11 per 100,000 population; 95% UI: 941.16–1453.17), Mali (1127.77 per 100,000 population; 95% UI: 859.54–1475.74), Iran (1122.96 per 100,000 population; 95% UI: 903.66–1377.07), and Guinea (1112.56 per 100,000 population; 95% UI: 838.61–1454.62). For further details, refer to Additional File 1.

### 4.3 Differences in the burden of glaucoma by gender and five-year age groups

**Figure 3:**
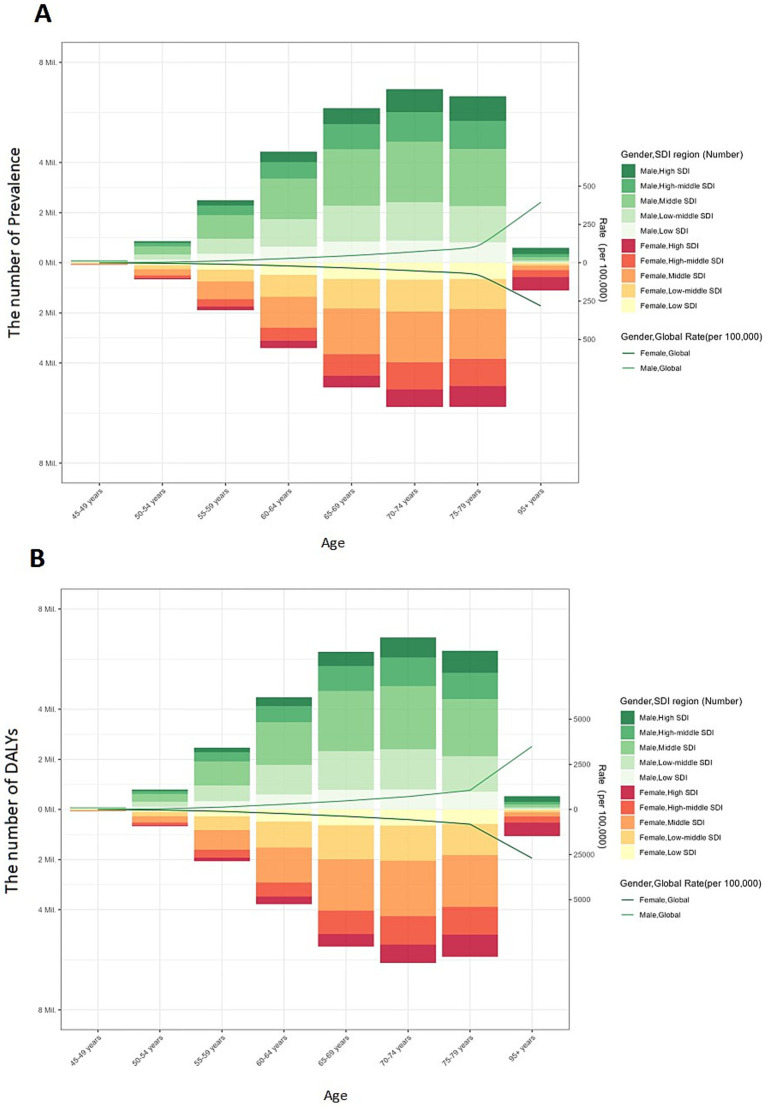
Global burden of glaucoma prevalence and DALYs by Five-Year Age Intervals, Gender, and SDI Regions. The number and age-standardized prevalence **(A)** and DALYs **(B)** in 2019 reported by sex, age, SDI region. DALYs: disability-adjusted life-years; SDI: sociodemographic index.

In the 2021 study, as illustrated in Figure 3, we observed that the global prevalence of glaucoma and DALYs significantly increased with age. Additionally, DALYs were generally higher in males than in females. Although the difference in the number of cases between genders was small—7,745,759 cases in males compared to 7,574,625 cases in females—the total DALYs for males (803,531.7) were notably higher than for females (732,069.6), indicating a gender disparity in disease burden.

This gender disparity in prevalence and DALYs becomes more evident with age, particularly among older age groups. For males, both prevalence and DALYs peaked in the 70–74 age group. In contrast, for females, prevalence peaked in the 70–74 age group, while DALYs peaked in the 80–84 age group. As a result, the global burden of glaucoma is more significant in the male population.

### 4.4 Cross-national inequalities in eye health

**Figure 4:**
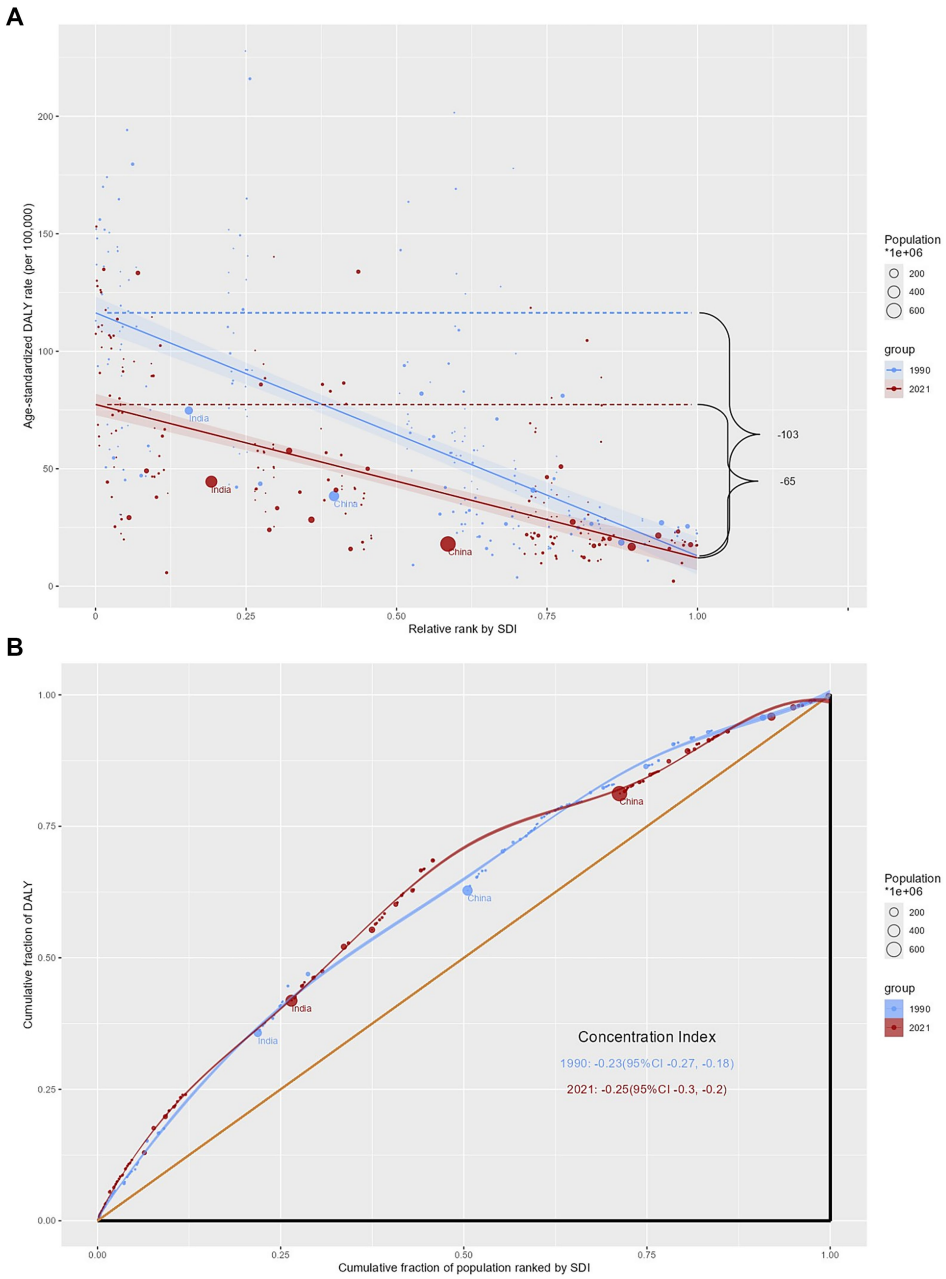
Cross-National Inequalities in glaucoma. Health inequality regression curves **(A)** and concentration curves **(B)** for the prevalence of glaucoma from 1990 to 2021 across the world. DALYs: disability-adjusted life-years; SDI: sociodemographic index.

Among the 204 countries analyzed in Figure 4A, significant and relative inequalities in the burden of glaucoma related to the SDI are evident, with low SDI countries experiencing a disproportionately higher burden. In 1990 and 2021, the SII was −103 (95% CI: −118–−89) and −65 (95% CI: −75–−55) DALYs per 100,000 population, respectively, indicating a negative correlation between ASDR and per capita GDP. This reduction suggests that the inequality in age-standardized glaucoma burden between high-income and low-income countries has decreased over this period. For further information, see Additional File 2.

In contrast, the relative inequality analysis in Figure 4B indicates that the CI remained nearly unchanged from 1990 (−0.23, 95% CI: −0.27–−0.18) to 2021 (−0.25, 95% CI: −0.30–−0.20).

### 4.5 Frontier analysis on the basis of age-standardized DALYs

**Figure 5:**
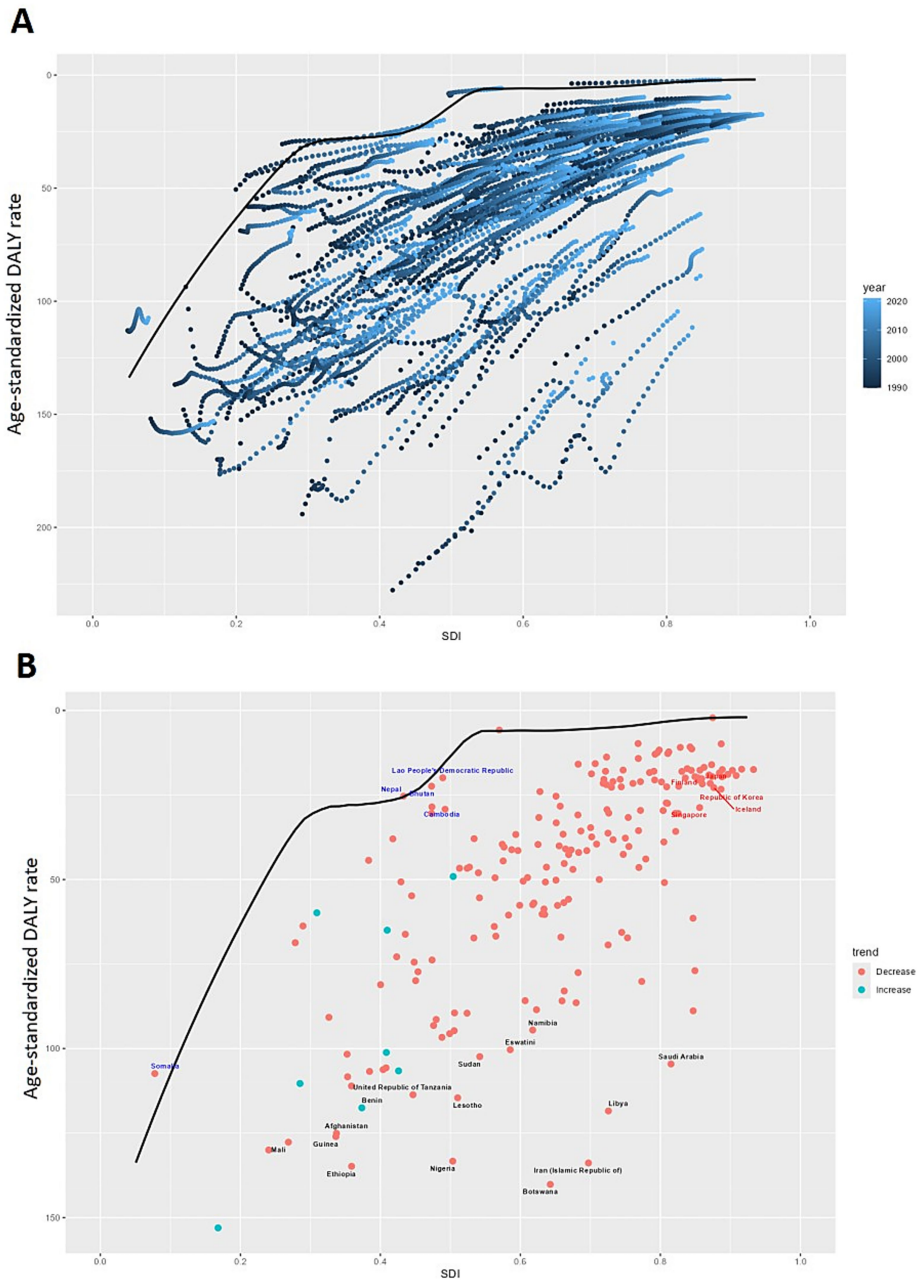
Frontier analysis of glaucoma Based on DALYs. Frontier analysis (represented by the solid black lines) explores the relationship between the Socio-Demographic Index (SDI) and age-standardized DALYs for glaucoma. The color gradient in **(A)** illustrates the progression of years, ranging from light shades representing 1990 to the darkest shades denoting 2021. **(B)** Each dot signifies a specific country or territory for the year 2021, with the top 15 countries displaying the most significant deviation from the frontier labeled in black. Countries with low SDI (> 0.455) and minimal deviation from the frontier are highlighted in blue, while those with high SDI (> 0.805) and notable deviation for their developmental level are emphasized in red. The direction of change from 1990 to 2021 in ASR is indicated by the color of the dots: decrease dots represent a decrease, while increase dots signify an increase.

In assessing the performance of DALYs based on the development status of countries or regions, we used data from 1990 to 2021 and conducted a frontier analysis of ASDR and the SDI (see Figure 5A). The boundary line defines the potential ASDR achievable based on the SDI. The actual gap, represented by the distance from the boundary, indicates the disparity between observed and potentially achievable ASDR, considering the SDI of the country or region.

Overall, countries with a higher SDI observed the smallest efficiency gaps; however, countries with a medium SDI showed the largest efficiency gaps. Botswana, Ethiopia, Nigeria, Iran, and Mali are the top five countries with the most potential to narrow this gap. Notably, the leading countries in performance are not limited to developed nations—two low-SDI countries, Nepal and Bhutan, have very small efficiency gaps, whereas several high-SDI countries, including Singapore, South Korea, Iceland, Finland, and Japan, exhibited larger efficiency gaps than expected based on their SDI (see Figure 5B and Additional File 3).

### 4.6 Global glaucoma prevalence and DALY projections

**Figure 6:**
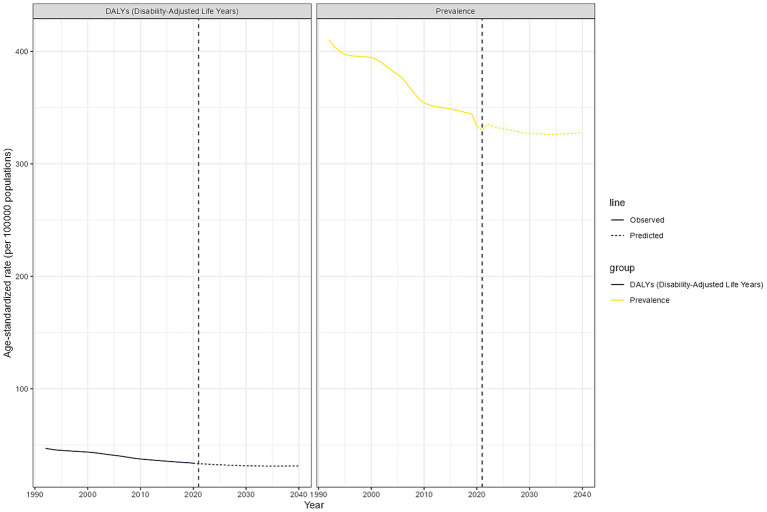
Global glaucoma prevalence and DALY projections. Age-standardized DALY and prevalence of global glaucoma during the observation period (1990–2021) and forecast period (2022–2040).

An age-period-cohort analysis based on Nordpred was used to estimate global trends in glaucoma prevalence and DALYs through 2040. As shown in Figure 6, the burden of glaucoma is expected to gradually decrease from 2021 to 2040. During this period, the ASR of prevalence are projected to decrease by 2.23, and the ASRs of DALYs are expected to decrease by 1.89. By 2040, the global ASPR and ASDR for glaucoma are projected to be 329.37 and 31.24, respectively (Figure 6, Additional File 4).

## 5 Discussion

In this study, we selected two outcome indicators—prevalence and DALYs—to analyze glaucoma burden trends over the past 32 years across 204 countries and regions. Compared with GBD 2019, the GBD 2021 study incorporated additional research and expanded data sources, providing the most up-to-date estimates of glaucoma epidemiology and enhancing data reliability. Over this period, the global numbers of prevalence and DALYs increased by 86.24 and 62.51%, respectively. However, the AAPC for ASPR and ASDR was negative globally and across all 21 GBD regions, indicating an overall declining trend. Despite rising absolute case numbers driven by population growth and aging, advances in diagnostic techniques and treatment have substantially improved disease management. For example, the adoption of optical coherence tomography (OCT) has enabled earlier detection and intervention, while newer intraocular pressure–lowering medications and minimally invasive surgeries have enhanced disease control. Global health initiatives, such as the WHO “Vision 2020” project, have also expanded access to eye care services in low- and middle-income countries, contributing to the global decline in prevalence and ASDR (15, 16).

The COVID-19 pandemic, however, posed short-term challenges. Routine eye examinations were often postponed, and access to diagnostic tests and surgical treatments was disrupted, leading to delayed diagnoses and disease progression. Medication supply chain interruptions further affected management. Although our results suggest that the long-term global trend in glaucoma burden remains downward, the pandemic likely exacerbated disparities, particularly in low-resource settings where healthcare systems were already fragile. Continued monitoring of its long-term impact is necessary to ensure continuity of glaucoma care during future global health crises (17).

Regional differences remain striking. In the 2021 GBD study, the highest ASPR of glaucoma was observed in Oceania, Central Europe, high-income North America, and sub-Saharan Africa, while the lowest occurred in Central Latin America, North Africa and the Middle East, and South Asia. The highest ASDR was recorded in Western and Eastern sub-Saharan Africa, exceeding 100 cases per 100,000 population, whereas Southern Latin America and Southeast Asia reported the lowest levels. These disparities reflect the interplay of socioeconomic, healthcare, and biological factors. High-income countries benefit from strong healthcare systems, advanced diagnostics, and wider access to treatment, resulting in lower disease burden. In contrast, low- and middle-income countries face limited infrastructure, shortages of ophthalmologists, delayed diagnosis, and financial barriers, leading to higher burden. Additionally, biological factors such as thinner corneal thickness and structural variations in the trabecular meshwork contribute to the higher prevalence of primary open-angle glaucoma (POAG) in African populations, reinforcing the need for targeted interventions in sub-Saharan Africa (18–22).

Sex and age also influence disease burden. We observed that in males, glaucoma prevalence and DALYs peaked at ages 70–74, while in females, prevalence peaked at the same age but DALYs peaked later, at 80–84 years. This pattern may reflect earlier onset of severe impairment in men due to lifestyle factors such as smoking and alcohol use, whereas women experience slower progression but greater cumulative burden because of longer life expectancy and reduced estrogen protection after menopause. These differences highlight the importance of gender- and age-specific prevention and management strategies (23–26).

Our study further demonstrated that ASDR negatively correlates with SDI at both regional and national levels, underscoring the role of socioeconomic factors in shaping glaucoma burden. Although absolute inequalities in glaucoma burden have declined in recent decades, relative inequalities remain largely unchanged. Low- and middle-income countries continue to face disproportionate challenges due to weaker health systems, fewer trained specialists, and the economic impact of vision loss, which reduces labor productivity and perpetuates poverty. Addressing these issues requires strengthening health system capacity, improving access to affordable care, and expanding international cooperation, particularly in Africa and South Asia (27–29).

Frontier analysis revealed that countries at different development levels continue to exhibit considerable efficiency gaps, with many performing below the achievable minimum burden relative to their SDI. Encouragingly, some low-SDI countries achieve leading outcomes despite resource constraints, serving as models for optimizing glaucoma care in similar contexts. Life expectancy also plays a role in shaping glaucoma burden: populations with longer lifespans face greater cumulative risk and longer disease progression, leading to higher DALY rates, whereas shorter life expectancy may limit exposure to glaucoma but reflects broader health system weaknesses. Moreover, the high prevalence of normal-tension glaucoma in countries such as Japan and South Korea underscores the importance of genetic, environmental, and healthcare-seeking differences that shape national-level burdens (30–34).

Future research should prioritize longitudinal cohort studies to evaluate glaucoma progression under different socioeconomic contexts, as well as subtype-specific analyses (e.g., open-angle, angle-closure, and normal-tension glaucoma) to inform targeted strategies. Incorporating real-world data from electronic health records and population-based surveys could refine burden estimates, while continued evaluation of the long-term effects of global disruptions such as COVID-19 remains essential. These directions will strengthen the evidence base for developing tailored interventions and guiding equitable resource allocation.

Finally, despite persistent challenges, projections suggest that global glaucoma prevalence and age-standardized DALY rates will continue to decline until 2040. This encouraging trend reflects progress in prevention and management, supporting the wider adoption of effective measures worldwide. Nonetheless, caution is warranted in interpreting results, as limitations of the GBD database remain, including possible misclassification, lack of subtype-specific data, and regional variability in data quality. Future studies should address these gaps with more rigorous methodologies to ensure reliable and actionable evidence.

## 6 Conclusion

This study utilizes data from the 2021 GBD Study to offer a comprehensive analysis of the burden of glaucoma at global, regional, and national levels. The findings indicate that, although the prevalence and DALY associated with glaucoma have increased worldwide over the past few decades, both metrics are projected to decline from 2021 to 2040. This encouraging trend may be attributed to the gradual improvement in glaucoma prevention and management strategies globally.

The study also reveals significant regional disparities, particularly in areas with a low SDI, where the health burden of glaucoma remains high. This uneven distribution highlights both challenges and opportunities for optimizing health outcomes in resource-limited settings. The findings emphasize the need for global health policymakers to consider regional inequalities in the glaucoma burden when developing control strategies and allocating resources. Future research should focus on analyzing countries that have achieved notable success in glaucoma prevention and treatment to identify and disseminate effective practices and policies.

Conversely, in countries where glaucoma management is less successful, in-depth analysis is required to uncover the root causes of these challenges and to develop tailored support and improvement strategies. By systematically analyzing these issues and sharing successful strategies, the global health community can more effectively work towards reducing the burden of glaucoma worldwide.

## Data Availability

The original contributions presented in the study are included in the article/supplementary material, further inquiries can be directed to the corresponding authors.

## Footnotes

^1^ http://ghdx.healthdata.org/gbd-results-tool
